# Synthesis and Biological Activity of a New Indenoisoquinoline Copper Derivative as a Topoisomerase I Inhibitor

**DOI:** 10.3390/ijms241914590

**Published:** 2023-09-26

**Authors:** Caroline Molinaro, Nathalie Wambang, Sylvain Pellegrini, Natacha Henry, Marc F. Lensink, Emmanuelle Germain, Till Bousquet, Jérôme de Ruyck, Katia Cailliau, Lydie Pélinski, Alain Martoriati

**Affiliations:** 1 Univ. Lille, CNRS, UMR 8576-UGSF-Unité de Glycobiologie Structurale et Fonctionnelle, F-59000 Lille, France; caroline.molinaro.fr@gmail.com (C.M.); marc.lensink@univ-lille.fr (M.F.L.); jerome.de-ruyck@univ-lille.fr (J.d.R.); katia.cailliau-maggio@univ-lille.fr (K.C.); 2 Univ. Lille, CNRS, Centrale Lille, Univ. Artois, UMR 8181-UCCS-Unité de Catalyse et Chimie du Solide, F-59000 Lille, France; wnath18@yahoo.fr (N.W.); sylvain.pellegrini@univ-lille.fr (S.P.); natacha.henry@univ-lille.fr (N.H.); till.bousquet@univ-lille.fr (T.B.); 3 Univ. Lille, Inserm U1003-PHYCEL-Physiologie Cellulaire, F-59000 Lille, France; emmanuelle.germain@univ-lille.fr

**Keywords:** indenoisoquinoline, copper(II) complex, topoisomerase I, adenocarcinoma, molecular modeling, DNA intercalation

## Abstract

Topoisomerases are interesting targets in cancer chemotherapy. Here, we describe the design and synthesis of a novel copper(II) indenoisoquinoline complex, **WN198**. The new organometallic compound exhibits a cytotoxic effect on five adenocarcinoma cell lines (MCF-7, MDA-MB-231, HeLa, HT-29, and DU-145) with the lowest IC_50_ (0.37 ± 0.04 μM) for the triple-negative MDA-MB-231 breast cancer cell line. Below 5 µM, **WN198** was ineffective on non-tumorigenic epithelial breast MCF-10A cells and *Xenopus* oocyte G2/M transition or embryonic development. Moreover, cancer cell lines showed autophagy markers including Beclin-1 accumulation and LC3-II formation. The DNA interaction of this new compound was evaluated and the dose-dependent topoisomerase I activity starting at 1 μM was confirmed using in vitro tests and has intercalation properties into DNA shown by melting curves and fluorescence measurements. Molecular modeling showed that the main interaction occurs with the aromatic ring but copper stabilizes the molecule before binding and so can putatively increase the potency as well. In this way, copper-derived indenoisoquinoline topoisomerase I inhibitor **WN198** is a promising antitumorigenic agent for the development of future DNA-damaging treatments.

## 1. Introduction

Metal-based drugs have been designed and developed for their cytotoxic effects on cancer cells since the discovery of platinum’s antitumor properties [1,2,3,4]. As they adopt a variety of coordination geometries, essential trace metals [5,6] are used for the implementation of metal-based complexed drugs in anticancer chemotherapies. In particular, numerous developed copper complexes have highly efficient antitumor activity [7,8,9] associated with various action mechanisms ranging from chelators [10], ionophores [11], and proteasome inhibitors [12] to inhibition of topoisomerase I and/or topoisomerase II that results in severe DNA damage, cell cycle arrest, and ultimately cancer cell death [13,14,15].

**Figure 1:**
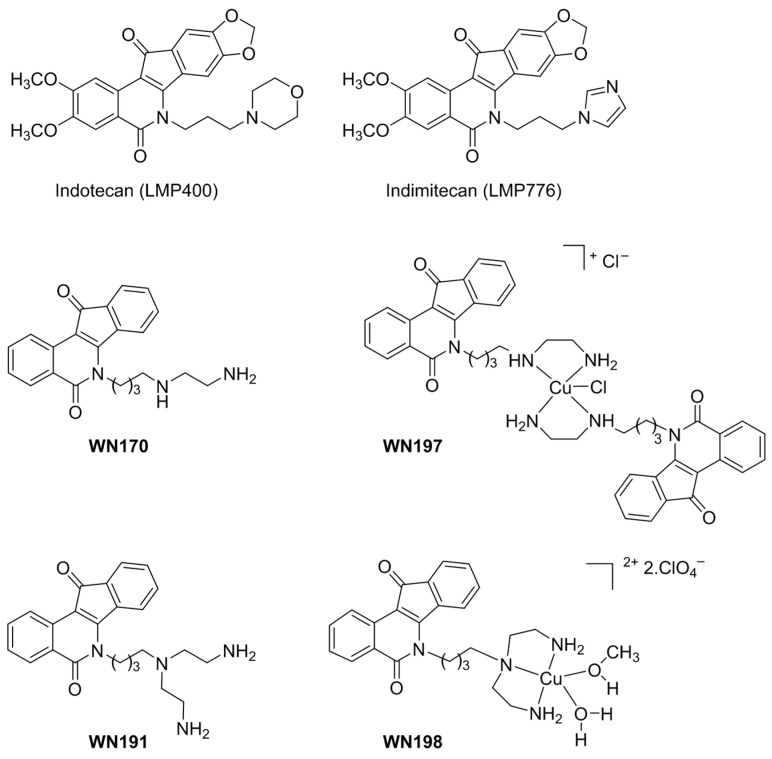
Biologically active indenoisoquinolines.

Novel topoisomerase I inhibitors, named indenoisoquinolines [16,17,18,19], were identified to have greater chemical stability compared to camptothecin and its derivatives currently used as a second-line treatment against endocrine-resistant breast cancer. The indenoisoquinoline derivatives indotecan (LMP400) and indimitecan (LMP776) have completed phase I/II clinical trials (Figure 1).

In previous studies, we have shown that copper complex **WN197**, derived from **WN170**, could be a new efficient drug to counteract cancer cells at low doses and exerts a specific cytotoxic effect at low concentration (IC_50_ of 0.5 μM) on three adenocarcinoma cell lines from breast, cervix, and colon but not on non-cancerous breast MCF-10A cells and chemo-resistant pulmonary cancer H69AR cells (Figure 1) [15]. To our knowledge, no studies on the presence of branching on the indenoisoquinoline side chain have been reported. Our interest in the search for anticancer organometallic drugs has been focused on the synthesis of a novel triamine ligand **WN191** and its corresponding tridentate Cu(II) complex **WN198** (Figure 1). The DNA interaction of this new compound was evaluated and its cytotoxicity was tested on adenocarcinoma cell lines, revealing the lowest IC_50_ for the triple-negative MDA-MB-231 breast cancer cell line.

## 2. Results

### 2.1. Synthesis and Characterization

**Figure 2:**
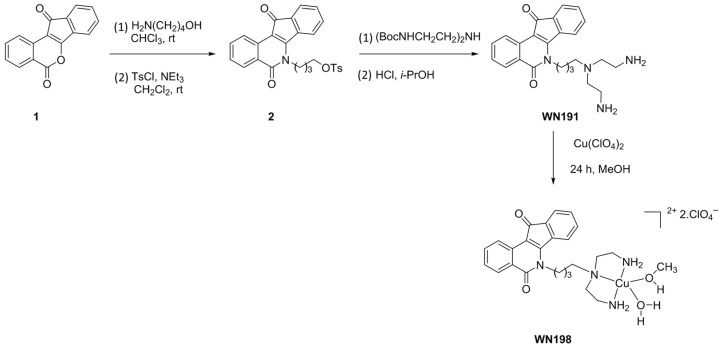
Synthesis of the ligand **WN191** and the Cu(II) complex **WN198**.

The synthesis of the ligand **WN191** and its corresponding copper complex **WN198** are described in Figure 2. Indenoisoquinoline **WN191** was first obtained in a four-step reaction. Condensation of the commercially available benzo[*d*]indeno [1,2-*b*]pyran-5,11-dione **1** with a primary aminoalcohol was followed by tosylation of the alcohol function to lead to compound **2** in 85% yield. The substitution of the tosyl group by the diprotected triamine and the deprotection of the Boc group led to the indenoisoquinoline derivative **WN191** in 88% yield. Their complexation by copper(II) perchlorate in methanol afforded the copper indenosisoquinoline **WN198** in 70% yield.

**Figure 3:**
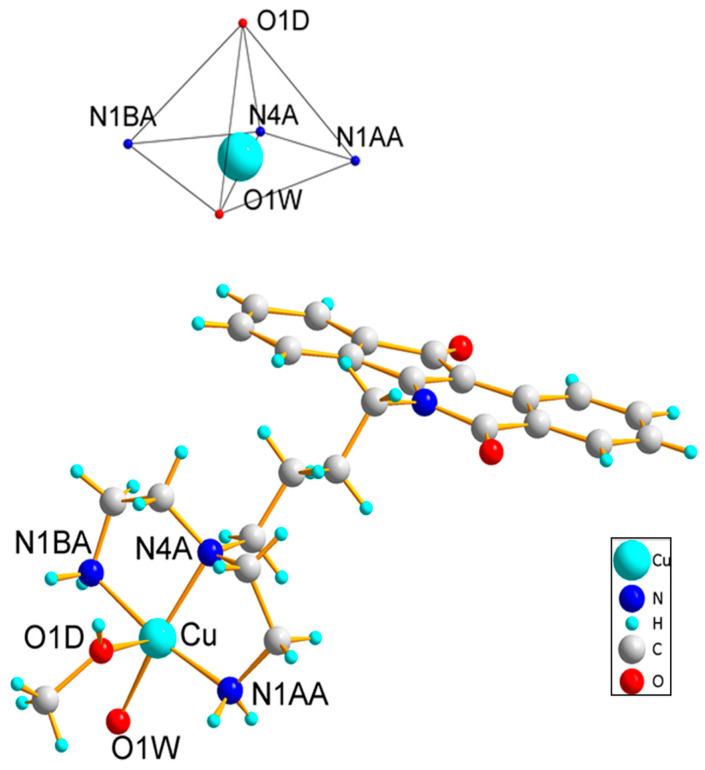
Molecular structure of Cu complex **WN198** showing the local geometry around the copper and ligand. Selected bond lengths (Ǻ) and angles (°): Cu-N1BA 1.998(8); Cu-N4A 2.014(6); Cu-N1AA 1.990(7); Cu-O1W 1.980(7); Cu-O1D 2.333(7); N1BA Cu N4A 85.1(3); N1AA Cu N4A 86.2(3); O1W Cu N1AA 94.5(3); O1W Cu N1BA 94.6(3); N1BA Cu O1D 91.2(4); N4A Cu O1D 101.9(3); N1AA Cu O1D 88.1(3); O1W Cu O1D 94.0(3).

The structure of **WN198** was established by single-crystal X-ray analysis. The drawing is displayed in Figure 3. The Cu complex crystallizes in the triclinic space group *P*1 with two formula units per unit cell. The XRD results show that the Cu(II) complex consists of a mononuclear [Cu(II)L(H_2_O)(CH_3_OH)]^2−^ (where L is the indenoisoquinolinetriamine ligand) unit and two (ClO_4_)^−^. The five coordinated Cu(II) ions occupy the center of the distorted square base of the pyramid consisting of three nitrogen atoms from the indenoisoquinolinetriamine ligand and one oxygen atom of a water molecule (1.995 Å). One O atom of (CH_3_)OH completes the pyramidal environment with an axial bond distance of Cu–O = 2.333 (7) Å.

### 2.2. ***WN191*** and ***WN198*** Display Cytotoxic Activity on Five Adenocarcinoma Cell Lines at Low Doses

Cell viability was assayed on breast cancer cells (MCF-7), triple-negative breast cancer cells (MDA-MB-231), cervix cancer cells (HeLa), colorectal cancer cells (HT-29), and prostate cancer cells (DU-145), Table 1. The IC_50_ values obtained for **WN191** were respectively 0.58 µM, 1.12 µM, 0.80 µM, 0.53 µM, and 1.09 µM. The copper metal significantly enhances the toxicity of the indenoisoquinoline on the triple-negative breast cancer line MDA-MB-231. For **WN198**, IC_50_ values were lower by a factor of 3.02 for MDA-MB-231 (0.37 µM), or close to a factor of 1.11 for HeLa (0.72 µM) and 1.04 for DU-145 (1.04 µM), or slightly higher by a factor 1.53 for breast cancer hormone-dependent MCF-7 (0.89 µM) and by a factor of 2 for HT-29 (1.06 µM). All IC_50_ values for compounds **WN191** and **WN198** were below the cisplatin values ranging from 2 to 40 µM. Compared to **WN170** [15], **WN198**’s IC_50_ value was lower by a factor of 2.36 for MDA-MB-231 but not for all the other cell lines.

**Figure 4:**
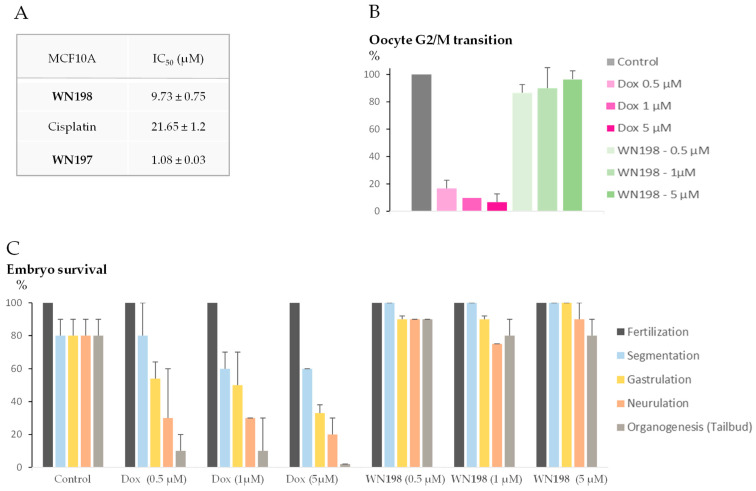
Non-cancerous cell viability under **WN198** treatment. (**A**) MCF-10A IC_50_ values are expressed as the mean ± SD of three independent experiments. Cisplatin and **WN197** were used as control. (**B**) *Xenopus* oocyte G2/M transition was scored by the determination of a white spot at the animal pole showing the progression from prophase I to metaphase II (meiosis maturation) 12 h after microinjection and balneation with corresponding drugs (0.5, 1, 5 µM) and external stimulation by progesterone as a natural inducer (4 µM). Experiments were performed on 10 to 20 oocytes from three different females. (**C**) The viability of *Xenopus* embryos was followed after incubation with corresponding drugs (0.5, 1, 5 µM). Stages were identified using the Nieuwkoop and Faber table [24]: segmentation, (3 h 30 min after fertilization), gastrulation (9 h after fertilization), neurulation (19 h 45 min after fertilization), tailbud (24 h after fertilization). Experiments were performed on two independent fertilizations and 10 to 40 embryos in each condition.

MTS viability assays were performed on the MCF-10A human non-tumorigenic epithelial cell line, most commonly used as a model for normal human breast cells (Figure 4A). The concentration of **WN198** required to inhibit 50% of the MCF-10A viability was significantly higher compared to the mean obtained from the other five adenocarcinomas’ IC_50_. **WN198**’s IC_50_ was smaller compared to cisplatin but higher than that of **WN197**, a copper complex and a topoisomerase inhibitor [15]. Additionally, we tested the ability of **WN198** to alter *Xenopus* oocyte maturation and embryonic development, two useful single and highly organized cell systems to test metals and chemical effects [20,21,22,23]. *Xenopus* oocytes undergo a G2/M transition, after progesterone stimulation and concomitant microinjection and balneation with **WN198** (0.5, 1, and 5 µM). At those doses, oocytes treated with doxorubicin, a topoisomerase II inhibitor, displayed an inhibition of their meiosis progression (Figure 4B). *Xenopus* embryos could survive **WN198** but not doxorubicin treatment. For 1 and 5 µM of doxorubicin, the percentage of surviving embryos was lowered at segmentation (cell division), and only half of the embryos survived gastrulation (involving cell mobility to build a three-layered organized embryo). For all doxorubicin concentrations tested, the percentage of live embryos was drastically lowered at the time of neurulation and organogenesis (Figure 4C).

### 2.3. ***WN191*** and ***WN198*** Are Concentration-Dependent Topoisomerase Inhibitors

**Figure 5:**
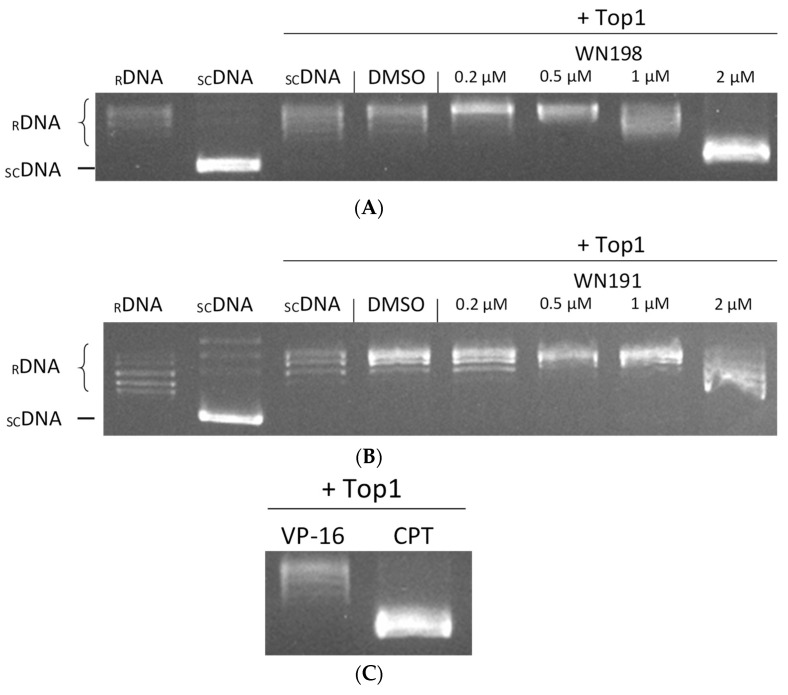
**WN191** and **WN198** inhibited human topoisomerase I activity in a dose-dependent manner. Topoisomerase I (Top1) activity is determined by in vitro assays after the addition of either (**A**) **WN198** or (**B**) **WN191** at different concentrations (0.2, 0.5, 1, and 2 µM, lanes 5–8). Relaxed DNA (_R_DNA, lane 1) or supercoiled DNA (_SC_DNA, lane 2) is used as migration control. The Top1 activity control allowing the relaxation of _SC_DNA is in lane 3. DMSO is the solvent control (5%, lane 4). _SC_DNA is used in all other reactions in the presence of Top1. (**C**) Etoposide (VP-16, 50 µM; topoisomerase II (Top2) poison, lane 1) is the negative control of Top1 activity inhibition, and camptothecin (CPT, 10 µM; Top1 poison, lane 2) the positive control of Top1 activity inhibition. After topoisomerase reactions, DNA is run in a 1% agarose gel, stained with ethidium bromide (0.5 µg/mL), and visualized under UV light.

To determine the effect of **WN191** and Cu(II) complex **WN198** on topoisomerase activity, in vitro tests were realized. Topoisomerase I assays rely on the relaxation of supercoiled DNA by an active topoisomerase I. With topoisomerase I, supercoiled DNA shows a relaxed profile (Figure 5). With a well-known topoisomerase I inhibitor, camptothecin (CPT), the DNA relaxation was disturbed and a part of the DNA remained supercoiled. With the addition of 1 and 2 µM of **WN198** in the reactional mixture, the quantity of relaxed DNA is decreased, indicating disruption of topoisomerase I activity. **WN191** disrupted topoisomerase I activity at 2 µM. DMSO (solvent control) and VP-16 (etoposide, a topoisomerase II inhibitor) have no effect on DNA relaxation induced by topoisomerase I, showing their lack of inhibitory effect on topoisomerase I activity.

### 2.4. ***WN198*** Intercalates in DNA

Melting curve and fluorescence measurements were performed to confirm the results shown in Table 2 and ascertain **WN191** and **WN198** intercalation in DNA.

The binding affinities, determined using a fluorescence-quenching assay based on DNA-binding competition between the intercalating drug ethidium bromide and the tested molecules, were used to gain insight into the DNA-binding affinity. The apparent DNA-binding constant Kapp value of the Cu(II) complex (10.791 ± 1.638 10^7^ M^−1^) is higher compared to the original ligand **WN191** value (8.964 ± 0.964 10^7^ M^−1^). The complexation of indenoisoquinoline ligand by copper allows a stronger interaction with DNA (Table 2). The EtBr displacement is in contrast only 2% different between the two compounds.

### 2.5. Modeling of the Interaction between ***WN170***, ***WN191***, and ***WN198*** and Top1

**Figure 6:**
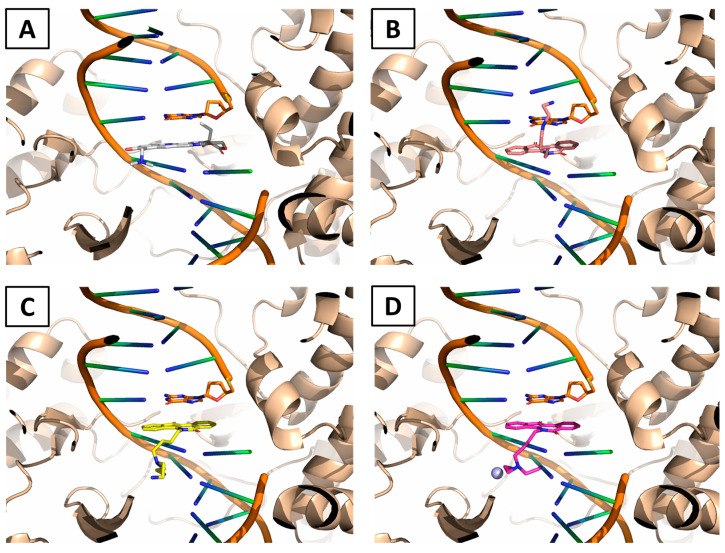
Interaction models of the studied ligands and Top1. (**A**) Crystal structure of the topotecan poison (grey sticks) fitting the DNA groove (orange sticks) of Top1 (brown helices) (PDB ID: 1K4T, 2.1 Å). (**B**) The binding mode of **WN170** (salmon sticks) is similar to topotecan’s binding mode. The main interaction is taking place inside the DNA groove. (**C**) The interaction model of **WN191** (yellow sticks) is slightly different from **WN170** as the larger branched arm has to switch down. The main part of the infarction is occurring through the aromatic ring. (**D**) The binding mode of **WN198** (pink sticks with the copper molecule as a blue ball) is similar to the binding mode of **WN191**.

As no experimental structures of complexes were available, we designed an approach to understand how our putative ligands could bind to Top1 and could intercalate DNA. The starting point was the crystal structure of human DNA Top1 in complex with topotecan poison (Figure 6A) (PDB ID: 1K4T, 2.1 Å). Based on the coordinates of the poison, we subsequently modeled the interaction of **WN170**, **WN191**, and **WN198** by molecular docking. The full procedure of molecular modeling is detailed in Section 4.7.

On one hand, based on these models, the previously described **WN170** adopts a similar conformation to the commercial poison topotecan which has an aromatic moiety intercalating the DNA (Figure 6B). The relatively small branched chain of **WN170** can easily fit the binding cavity. On the other hand, the complex between **WN191** and Top1 is similar to the previous one with the aromatic moiety totally fitting the DNA groove (Figure 6C). Nevertheless, one can observe that the larger branched arm of **WN191** had to switch to another position, putatively decreasing with a small impact on the affinity of this molecule. Moreover, **WN198** has a similar structure as **WN191** with a copper ion chelating a water molecule. This interaction was difficult to model by molecular mechanics as copper ions are not well parametrized in a classical forcefield. One model was proposed (Figure 6D) that can be easily superimposed on the ligand **WN191**. Although the binding modes of **WN191** and **WN198** are almost the same, their activities are slightly different, which may be explained by copper ion playing a role in the stabilization of the ligand before the binding to the DNA groove as discussed in Section 3.

### 2.6. ***WN198*** Induces Autophagy but Not Apoptosis

**Figure 7:**
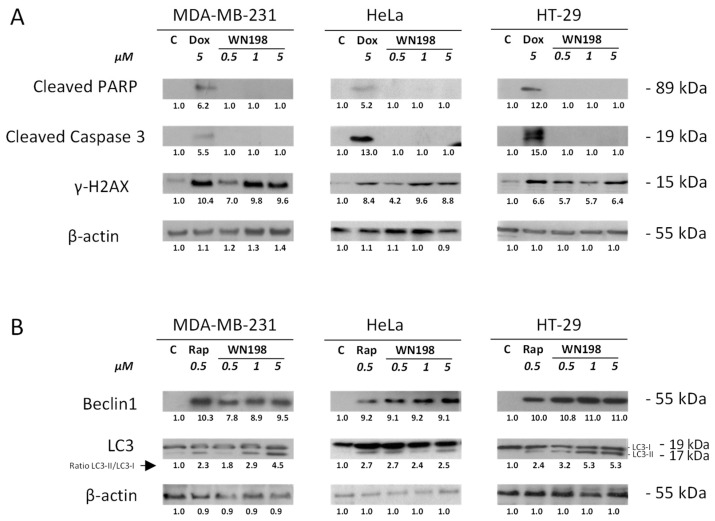
**WN198**-induced autophagy. MDA-MB-231, HeLa, and HT-29 cell lines were treated for 24 h with doxorubicin (Dox, 5 µM), rapamycin (Rap, 0.5 µM), or **WN198** (0.5, 1, 5 µM). (**A**) Western blots were performed with anticleaved caspase 3 and anticleaved PARP antibodies for detection of apoptosis. Anti-γH2AX antibody was used for detection of DNA breaks. (**B**) Western blot analysis of Beclin-1, and LC3 markers were used for detection of autophagy. LC3-II/LC3-I ratio was determined (arrow). β-actin level was used as a loading control. Relative protein levels were expressed by densitometry using software (Fiji Software, v1.52i).

Apoptosis can be activated after DNA damage [25,26]. However, the early and late apoptosis markers, respectively, cleaved caspase 3 and cleaved PARP, were not detected after treatments with **WN198** at all concentrations tested, in three adenocarcinoma cell lines, MDA-MB-231, HeLa, HT-29, in contrast to doxorubicin treatment (Figure 7A). γH2AX, an indicator of DNA breaks, was detected after treatment with **WN198** and doxorubicin, indicating that **WN198** and doxorubicin could induce DNA damage. Untreated cells showed lower γH2AX signals. We then determined whether **WN198** could induce autophagy. Several autophagy markers [14] were present after 24h of treatment with 0.5, 1, and 5 µM of **WN198** and rapamycin, an inhibitor of the mTOR pathway known to trigger autophagy [27]. Beclin-1 was synthesized, and LC3-II (LC3-I in association with phosphatidyl-ethanolamine) was increased while control untreated cells did not show these markers (Figure 7B).

## 3. Discussion

Topoisomerases are overexpressed in cancer cells which divide rapidly and have a high frequency of M phase in their cell cycle. This overexpression can be exploited by inhibition of topoisomerases by appropriate drugs that generate a high number of DNA breaks leading to cancer cell death [28,29,30]. Cells overexpressing topoisomerases have been shown to better respond to topoisomerase inhibitors [31,32].

Since the discovery of platinum’s anticancer properties, platinum-derived drugs have become a mainstay of cancer therapy [2,33]. Other metal-based drugs have been designed and developed [3,4], like transition metals from the d-block of the periodic table (groups 3 to 12) [1,34,35], as they adopt a wide variety of coordination geometries. Indeed, previous studies have demonstrated organometallic compounds composed of metallic atoms such as copper [9], iron (e.g., ferrocifen/ferroquine [1,36]), ruthenium (e.g., indenoisoquinoline [37] and various complexes [38]), or platinum (e.g., cisplatin [39]), which can be used as efficient anticancer drugs. Among them, copper allows a modification in complexed ligand backbones and better DNA affinity [11,40,41]. Some copper derivatives interact with DNA, enhancing DNA damage and antitumor activity [42]. Other copper complexes inhibit topoisomerase activity and result in DNA damage, cell cycle arrest, autophagy, and death in cancer cells [13,14,15]. Recently, a new class of topoisomerase inhibitors derived from indenoisoquinoline were developed and selected for their capacity to escape efflux transporters implied in cell resistance and for their high stability [16,43]. These indenoisoquinoline derivatives are in phase I/II clinical trials [16,43].

In an attempt to develop new efficient organometallic compounds, a copper(II) indenoisoquinoline complex named **WN198** was synthesized with a branched triamine moiety attached to the indenoisoquinoline core. In this compound, the triamine ligand copper(II) was inserted at the end of the four-carbon chain as a linker because it was previously shown this moiety enhances the DNA-binding affinity due to many hydrogen bonds [36,44,45]. The Cu(II) complex structure displays a mononuclear unit of indenoisoquinolinetriamine and two (ClO_4_)^−^. At the center of a distorted square with three nitrogen atoms from the indenoisoquinolinetriamine and one oxygen atom from water are five coordinated Cu(II) ions.

In vitro tests reveal that **WN198** inhibits topoisomerase I in a dose-dependent manner starting at 1 μM and with optimum efficiency at 2 μM, while the initial compound (**WN191**) only starts inhibiting topoisomerase I at 2 μM. Furthermore, **WN198** could induce DNA breaks detected by a γH2AX signal. Because H2AX phosphorylation correlates with DNA lesions it is used as a marker. When DNA lesions are triggered, the activation of the DNA damage response (DDR) pathway leads to the phosphorylation of histone H2AX on serine 139 (γH2AX) by phosphoinositide 3-kinase-related kinase family members [46,47].

We could further determine that **WN198** interacts with DNA with an apparent DNA-binding constant value higher by a factor of 1.2 compared to the original indenoisoquinoline ligand value, showing that the complexation of the ligand with copper allows a slight improvement in the interaction with DNA. A catalytic mode of inhibition could also occur through the intercalation of **WN198** into the DNA as demonstrated by the melting curves and the fluorescence measurements. Indenoisoquinolines are composed of a planar skeleton, and the high affinity of the copper(II) complex for DNA could be the result of the π–cation interaction between the atom of Cu(II) coordinated with ligands and the base pairs [48,49]. DNA intercalation of **WN198** could impede topoisomerase’s access to the DNA fixation sites as seen for other topoisomerase inhibitors, such as anthracyclines [50,51]. Due to a strong affinity for DNA duplexes, anthracycline compounds prevent topoisomerase binding to DNA [50,51]. Doxorubicin, one of the most effective chemotherapeutic drugs used against solid tumors in the treatment of several cancer types, displays a poison activity at low doses and an intercalating catalytic inhibitory action at high doses. Indenoisoquinoline copper derivative **WN197** also efficiently induced MDA-MB-231, HeLa, and HT-29 cell death below 0.5 µM. The planar indenoisoquinoline skeleton of **WN197** displays high intercalation into DNA [15]. Low doses of **WN197** inhibit topoisomerases while, at higher doses, the compound has DNA intercalation properties. However, **WN197** exhibits two complexed ligand backbones around a single Cu(II) atom, compared to a single one in **WN198**. While some of the topoisomerase I inhibitors developed do not use intercalation [52], and knowing DNA-binding and topoisomerase I poisoning activities can be viewed as separate mechanisms [53], our results nevertheless encourage the synthesis of series of indenoisoquinoline drugs composed of a backbone with a four-carbon side chain around a metallic center to keep topoisomerase inhibition and strong DNA interaction properties.

Based on existing structures, we demonstrate by molecular modeling that our drugs interfere with DNA and thus block the activity. Thanks to the structural approach, we can clearly see that the most important moiety is the aromatic intercalating one.

The viability assays showed that low doses between 0.37 and 1.6 μM could induce cell death in breast, cervix, colon, and prostate cancer cell lines, from five highly prevalent adenocarcinomas. The lowest IC_50_ values were obtained for cervix and breast triple-negative cancer cell lines at doses below that of the original compound (respectively, by a factor of 1.1 and 3). These IC_50_ values are under the values determined for many other topoisomerase I inhibitors [9]. Higher doses were necessary to affect the MCF-10A human non-tumorigenic epithelial breast line or *Xenopus* oocyte G2/M transition and embryonic development. The IC_50_ of **WN198** on MCF-10A cells was higher compared to **WN197**, respectively, 9.73 and 1.080 µM, which makes **WN198** a better candidate to avoid side effects of chemotherapies on non-cancerous cells. The toxicity characteristics of **WN198** need further determination with respect to membrane permeability and the determination of cellular uptake.

Copper complexes or topoisomerase inhibitors arrest the cancer cell cycle in different phases and trigger cell death differently by apoptosis or senescence or have bimodal action through both apoptosis and autophagy [54,55,56]. Recently, another copper complex derived from the topoisomerase I inhibitor indenoisoquinoline was shown to arrest the cell cycle in the G2 phase through inhibition of Cdc25C phosphatase necessary to activate MPF (Cdk1/cyclin B) and to trigger cell death by autophagy [9,14,15]. **WN198** triggers autophagy, shown by the accumulation of Beclin-1 and the formation of LC3-II, but no apoptosis as cleaved markers such as PARP and caspase 3 were not detected in the cell lines MDA-MB-231, HeLa, and HT-29 for doses of **WN198** equal to and above the IC_50_.

The influence of indenoisoquinoline amine complexations with other metals should be examined in future studies with the knowledge that low minimum and necessary doses of chemotherapeutic compounds could be useful in circumventing normal cell death and limiting cardiotoxicity, a strategy employed for anthracyclines [57,58].

## 4. Materials and Methods

### 4.1. Chemistry

All commercial reagents and solvents were used without further purification. Cisplatin was procured from Alfa Aesar (Heysham, UK); DMSO from Sigma-Aldrich (Saint-Quentin-Fallavier, France). Stock solutions were prepared in DMSO. Melting points were determined with a Barnstead Electrothermal (BI 9300) capillary melting point apparatus and are uncorrected. The ^1^H and ^13^C NMR spectra were recorded on a Bruker AC300 spectrometer at 300 and 75.5 MHz, respectively, using tetramethylsilane (TMS) as internal standard and DMSO-d6 as solvent. Elemental analyses were performed with a vario MICRO element analyzer. Thin layer chromatography (TLC) was carried out on aluminum-baked Macherey-Nagel silica gel 60. Column chromatography was performed on silica gel (230–400 mesh). The electronic absorption spectra were acquired on a SPECORD^®^ PLUS UV–Vis double-beam spectrophotometer (Analytik Jena GmbH, Jena, Germany)). The molar conductance measurement was carried out using a CDRV 62 Tacussel electronic bridge, employing a calibrated 10^−2^ M KCl solution and 10^−3^ M solutions of compounds in DMSO. Purities of all tested compounds were ≥95%, as estimated by HPLC analysis. The high-resolution mass spectrum (HR-MS) was measured by REALACAT, University of Lille on a Synapt G2Si (Waters SAS, Saint-Quentin-en-Yvelines) equipped with an ion mobility cell, recorded in positive ion mode with an electrospray ionization (ESI) source.

#### 4.1.1. Synthesis of **WN191**

- 4-(5,11-Dioxo-5,11-dihydro-6*H*-indeno[1,2-*c*]isoquinolin-6-yl)butyl-4-methylbenzene sulfonate [59].

4-Aminobutan-1-ol (8 mmol, 0.74 mL) was added to a solution of indenopyranedione 1 (1.0 g, 4.0 mmol) in CHCl_3_ (40 mL). After stirring at room temperature for 18 h, the reaction mixture was diluted in CHCl_3_ (100 mL) and subsequently washed with distilled water (2 × 50 mL), HCl 0.1 M (1 × 25 mL), and brine (1 × 50 mL), dried over MgSO_4_, filtered, and concentrated in vacuo to afford dark-orange needles (1.2 g, 96%). To the crude product in CH_2_Cl_2_ (8 mL) was added TsCl (7.8 g, 4.1 mmol). Triethylamine (1.5 mL, 11 mmol) was added and the mixture was stirred at room temperature for 16 h. The resulting mixture was diluted with CH_2_Cl_2_ (50 mL) and washed with distilled water (2 × 20 mL) and then brine (20 mL). The organic layer was dried over Na_2_SO_4_ and concentrated. Purification by flash column chromatography on silica gel (CH_2_Cl_2_/MeOH, 99:1, as eluent) afforded the desired product **2** as a dark-red solid (1.5 g, 85%). Mp: 154 °C. ^1^H NMR (300 MHz, CDCl_3_) *δ* 8.60 (dd, *J* = 7.2, 0.3 Hz, 1H), 8.26 (dd, *J* = 6.8, 0.5 Hz, 1H), 7.75 (d, *J* = 8.3 Hz, 2H), 7.69 (td, *J* = 6.9, 1.3 Hz, 1H), 7.59 (dd, *J* = 6.1, 0.6 Hz, 1H), 7.45 (m, 4H), 7.30 (d, *J* = 7.9 Hz, 2H), 4.48 (t, *J* = 7.2 Hz, 2H), 4.13 (t, *J* = 5.6 Hz, 2H), 2.40 (s, 3H), 1.90 (m, 4H). ESI-MS (*m*/*z*): found: 474.1382 [M + H]^+^, calculated: 474.1375 [M + H]^+^.

- 6-(4-(Bis(2-aminoethyl)amino)butyl)-5*H*-indeno[1,2-*c*]isoquinoline-5,11(6*H*)-dione **WN191**

Compound 2 (800 mg, 1.7 mmol) was added to a solution of di-*tert*-butyl(azanediylbis(ethane-2,1-diyl))dicarbamate (2.6 mg, 8.4 mmol) in acetonitrile (25 mL). The resulting mixture was heated at 80 °C for 16 h. The solvent was evaporated in vacuo. The residue was taken up in CH_2_Cl_2_ (50 mL), washed with brine (2 × 25 mL), dried over MgSO_4_, and concentrated in vacuo. Purification by flash column chromatography on silica gel (CH_2_Cl_2_/EtOAc, 7:3, as eluent) afforded the *N*-protected product as a red solid (663 mg, 65%). Mp: 94 °C. 1H NMR (300 MHz, CDCl_3_) *δ* 8.79 (d, *J* = 7.8 Hz, 1H), 8.35 (d, *J* = 8.7 Hz, 1H), 7.72 (td, *J* = 6.9, 1.7 Hz, 1H), 7.64 (d, *J* = 6.5 Hz, 1H), 7.48–7.38 (m, 4H), 5.37 (m, 2H), 4.52 (t, *J* = 7.9 Hz, 2H), 3.18 (m, 4H), 2.55 (m, 6H), 1.98–1.18 (m, 2H), 1.70–1.65 (m, 2H), 1.36 (s, 18H). To a solution of protected amine (663 mg, 1.1 mmol) in CHCl_3_ (5 mL) was slowly added a solution of HCl 5M in 2-propanol (40 mL) at 0 °C. After stirring at room temperature for 18 h, the mixture was neutralized by KOH, extracted with CH_2_Cl_2_ (3 × 25 mL), dried over MgSO_4_, and concentrated in vacuo. Purification by recrystallization (Et_2_O/EtOH) afforded compound **WN191** as an orange solid (394 mg, 88%). Mp: 172 °C. ^1^H-NMR (300 MHz DMSO-d6) *δ* 8.41 (d, *J* = 8.2 Hz, 1H), 8.08 (d, *J* = 8.2 Hz, 1H), 7.72–7.60 (m, 2H), 7.52–7.35 (m, 4H), 4.34 (t, *J* = 8.9 Hz, 2H), 2.85 (m, CH_2_, 4H), 2.45 (m, 6H), 1.74 (q, *J* = 7.4 Hz, 2H), 1.53 (q, *J* = 8.9 Hz, 2H). ^13^C NMR (75 MHz DMSO-d6) *δ* 192.5, 1623, 156.1, 136.5, 134.8, 134.0, 133.9, 131.9, 131.7, 131.2, 128.0, 126.9, 106.8, 53.5, 53.3, 43.9, 31.5, 29.3, 28.9, 27.3, 26.7, 23.3. ESI-MS (*m*/*z*): found: 405.2279 [M + H]^+^, calculated: 405.2285 [M + H]^+^.

#### 4.1.2. Synthesis of Cu(II) Complex **WN198**

To a solution of **WN191** (298 mg, 0.74 mmol) in MeOH (8 mL) was slowly added a solution of Cu(ClO_4_)_2_.6H_2_O (273 mg, 0.74 mmol) in MeOH (7 mL). After stirring at room temperature for 24 h, the mixture was filtered and washed with methanol. The powder was recrystallized in a mixture of methanol/ether to give orange crystals (367 mg, 70%). Anal. calc. for C_25_H_34_Cl_2_CuN_4_O_12_: C, 41.88; H, 4.78; N, 7.81; found: C, 41.61; H, 4.60; N, 7.90. UV-vis in DMSO-H_2_O (19/01) 5.10 at 5.0 M, [λmax, nm (ε, M^−1^ cm^−1^)]: 659 (200), 460 (2980), 372 (12320), 349 (11920), 327 (11120). 1.21. Λm (Ω^−1^.cm^2^.mol^−1^): 51. IR (cm^−1^): 3326 (m) νas (NH_2_), 3269 (m) νs (NH_2_), 1656 (m) ν (C = O), 1548 (s) ν (C = C), 1077 (s), 622 (m) ν (ClO_4_^−^), 562 (s) ν (Cu-N). ESI-MS (*m*/*z*): found: 467.1507 [M − (H_2_O + MeOH)]^+^, calculated: 467.1508 [M − (H_2_O + MeOH)]^+^.

Crystal data, data collection, and structure refinement details are summarized in Table 3.

### 4.2. Cell Culture

Human cell lines from cervix cancer (HeLa), breast cancer (MCF-7), triple-negative breast cancer (MDA-MB-231), colorectal cancer (HT-29), prostate cancer (DU-145), and a human breast epithelial cell line, arguably the most commonly used normal breast cell model (MCF-10A), were obtained from the American Type Culture Collection (ATCC, Manassas, VA, USA). Cells were cultured at 37 °C in a humid atmosphere containing 5% CO_2_, in a DMEM culture medium supplemented with 10% fetal bovine serum (Dutscher, Dernolsheim, France), 1% Zell Shield (Dutscher), and 1% non-essential amino acids (Lonza, Basel, Switzerland). MCF-10A cells were maintained in MEBM (Lonza) supplemented with MEGM (Lonza) and 1% Zell Shield.

### 4.3. Cell Viability Assay

Cell viability was determined using the CellTiter 96^®^ AQ_ueous_ One Solution Cell Proliferation Assay (MTS test, Promega, Charbonnières-les-Bains, France). Cells were seeded in 96-well plates at a density of 2.10^3^ for 24 h before treatment with 0 to 100 µM of **WN198**, **WN191**, **WN170**, **WN197**, or cisplatin for 72 h. Cells were incubated for 2 h with 20 µL of CellTiter solution at 37 °C in 5% CO_2_, and the production of formazan from reduced 3-(4,5-dimethylthiazol-2-yl)-5-(3-carboxymethoxyphenyl)-2-(4-sulfophenyl)-2H-tetrazolium (MTS) was measured at 490 nm (SPECTROstar Nano, BMG LABTECH, Ortenberg, Germany). GraphPad Prism V6.0 software served to calculate IC_50_. Statistical differences between **WN198** and **WN191** were ascertained by a Student’s *t*-test (* *p* < 0.05, ** *p* < 0.001, *** *p* < 0.0005 and **** *p* < 0.0001).

### 4.4. Human Topoisomerase I In Vitro Activity

Topoisomerase I activity was determined with in vitro drug screening kits (TopoGEN, Inc., Buena Vista, CO, USA assays) based on the relaxation of supercoiled DNA into relaxed DNA as previously described [15]. Briefly, the assembled reaction mixture was composed of supercoiled pHOT1 DNA (250 ng), 10× TGS buffer (10 mM Tris-HCl pH 7.9, 1 mM EDTA), human topoisomerase I (5 units), the tested compound, and H_2_O to a final adjusted volume of 20 µL. **WN191** and **WN198** were tested at concentrations ranging from 0.2 to 2 µM. Camptothecin (10 µM) was used as a positive control (poison inhibitor of topoisomerase I activity), etoposide (100 µM) as negative control (inhibitor of topoisomerase II activity), 1% DMSO alone as vehicle control, and relaxed pHOT1 DNA (100 ng) alone as migration control. All reaction products were submitted to 1% agarose gel electrophoresis with EtBr (0.5 µg/mL) at 100 V for 1 h in TAE buffer (Tris-Acetate-EDTA; pH 8.3).

### 4.5. Ethidium Bromide Competition Test

Titrations of fluorescence were determined as described [36,45]. EtBr/**WN191** or EtBr/**WN198** with a molar ratio of 12.6/10 at concentrations ranging from 0.05 to 10 µM was used in a BPE buffer at pH 7.1. The excitation wavelength was 546 nm and the emission was monitored in the range of 560 to 700 nm (SPEX Fluorolog, Horiba-Jobin Yvon). EtBr displacement IC_50_ values were calculated with a fitting function incorporated into GraphPad Prism V6.0. The apparent binding constants were calculated using the equation K_app_ = (1.26 (K_app_(EtBr)/IC_50_) with K_app_(EtBr) = 10^7^ M^−1^.

### 4.6. Molecular Modeling

Receptor structure was the crystal structure of human DNA Top1 in complex with topotecan poison (Figure 4A) (PDB ID: 1K4T, 2.1 Å). All ligands and water molecules were removed except DNA. Simulation of the binding modes of the receptor and the designed compounds was performed using the GOLD docking program [60]. GOLD is based on a genetic algorithm and considered the ligand as flexible, while side chains of most residues were kept rigid. For the search procedure, a sphere of 30 Å was centered on the X, Y, and Z coordinates of the topotecan poison (Origin = 22.7 −9.4 26.9). Twenty conformations were generated for each ligand using default parameters. Then, all the different binding poses were scored with the ChemPLP scoring function. Subsequent energy minimization was performed using the AMBER forcefield. Finally, all the molecular representations were sketched using PyMOL [61].

### 4.7. Xenopus laevis Oocyte and Embryo Handling

*Xenopus laevis* females were obtained from the CRB-University of Rennes I, France. They were housed at PHExMAR, University of Lille. *Xenopus* were maintained in accordance with the EU Directive 2010/63/EU and the French National Guidelines for Use of Animals for Scientific Purposes. The experimental protocols were approved by the “Comité d’Ethique CEEA-75 en Experimentation Animale Nord-Pas de Calais-France” (*Xenopus* project number: F59-00913).

Females were anesthetized by immersion in a solution of Tricaine (MS222, Sandoz) at 3 g.L^−1^ for 1 h. The ovaries were surgically removed and placed in ND96 medium (96 mM NaCl, 2 mM KCl, 1.8 mM CaCl_2_, 1 mM MgCl_2_, 5 mM HEPES, pH 7.5) at 19 °C. Stage VI oocytes were harvested by using a 1 h collagenase A treatment (1 mg/mL, Boehringer Mannheim) for 45 min with a manual dissociation under a binocular microscope. Microinjections were performed under a binocular microscope with a Nishiryo positive displacement digital micropipette, in the equatorial region of the oocytes, with 60nl of control DMSO 0.1%, doxorubicin, or **WN198** at 0.5, 1, and 5 µM. Meiotic resumption was triggered by the addition of progesterone (4 µM) after microinjection and the addition of a corresponding concentration of doxorubicin or **WN198** to the external medium (0.5, 1, and 5 µM). The appearance of a white spot, resulting from the migration of the germinal vesicle at the apex of the pigmented hemisphere of the oocyte, indicated the meiosis process or G2/M transition. Oocytes arrested in prophase I progress to metaphase II and arrest [22,23,62].

For spawning, females were stimulated by a dorsal lymph sac injection of human chorionic gonadotropic hormone (900 U). Mature oocytes were obtained by a slight pressure on the animal’s ovaries. Testicles were surgically removed from a male after anesthesia in 5 g.L^−1^ MS222. In vitro fertilization was performed under gentle agitation in 0.22 µm filtered water with doxorubicin or **WN198** at 0.5, 1, and 5 µM or not. Eggs were deganguated with 2% L-cysteine with doxorubicin or **WN198** or not for 10 min and rinsed three times. Embryos were kept at 23 °C in daily renewed solution with doxorubicin or **WN198** or not. Embryo stages were identified using the Nieuwkoop and Faber table (Nieuwkoop and Faber, 1994) [24]: morula (segmentation, stage 6.5, 3 h 30 min after fertilization), gastrula (stage 10, 9 h after fertilization), neurula (stage 18, 19 h 45 min after fertilization), and tailbud (organogenesis, stage 22, 24 h after fertilization).

### 4.8. Electrophoresis and Western Blot

First, 7.5 × 10^5^ cells were seeded for 24 h before treatment with 5 µM doxorubicin (a positive control for apoptosis), 0.5 µM rapamycin (a positive control for autophagy), **WN198** (0.5, 1, 5 µM), or 0.1% DMSO (solvent control). After 24 h, cells were lysed in RIPA buffer (150 mM EDTA; 150 mM NaCl; 50 mM NaF, 1% Triton X-100; NP40 2%; 0.4% Na-deoxycholate; 0.6% SDS; 50 mM TRIS-HCl pH 4) supplemented with 1% protease cocktail inhibitors (Sigma-Aldrich) and phosphatase inhibitors (Roche SAS by Merck).

After centrifugation for 10 min at 12,000× *g*, the protein concentration of sample supernatants was determined using a Bradford assay (BioRad, Marnes-la-Coquette, France) at 595 nm (SPECTROstar Nano, BMG LABTECH). After denaturation at 75 °C for 10 min in 2× Laemmli buffer (65.8 mM TRIS-HCl pH 6.8; 26.3% glycerol; 2.1% SDS; 0.01% bromophenol blue; 4% β-mercaptoethanol, BioRad), 15 µg of samples was separated on 4–20% SDS PAGE gels (mini protean TGX, BioRad, Marnes-la-Coquette, France) for 1 h at 200 V in denaturing buffer (0.3% TRIS base; 0.1% SDS; 1.44% glycine). Proteins were wet transferred in 0.32% TRIS; 1.8% glycine; 20% methanol (Sigma-Aldrich, Saint-Quentin-Fallavier, France) onto a nitrocellulose membrane (Amersham Hybond, Dutscher, Bernolsheim, France) for 1 h at 100 V. After saturation with 5% low-fat dry milk in TBS with 0.05% Tween (Sigma-Aldrich), membranes were incubated overnight at 4 °C with primary antibodies: rabbit polyclonal antibodies were against cleaved caspase 3 or Beclin-1 (Cell Signalling Technology (CST, by Ozyme, Saint-Cyr-L’École, France), 1/1000), phosphorylated H2AX (S139, CST, 1/750), LC3 (CST, 1/50); goat polyclonal antibodies against β-actin (SCB, 1/1200); and cocktail antibodies against cleaved PARP (Abcam, Cambridge, UK, cell cycle and apoptosis cocktail, 1/1500). After three TBS-Tween washes of 10 min, membranes were incubated for 1 h with appropriate horseradish peroxidase-labeled secondary antibodies: antirabbit or antimouse (Invitrogen, 1/30,000) or antigoat antibodies (SCB, 1/30,000). After three 10 min TBS-Tween washes, the signals were revealed using a chemiluminescent assay (ECL Select, GE Healthcare, Dutscher, Bernolsheim, France) on hyperfilms (Amersham hyperfilm MP, Dutscher). β-actin was used as a loading control. Signals were quantified with ImageJ (Fiji Software, v1.52i) and normalized to respective loading controls.

## 5. Conclusions

A new copper(II) complex containing an indenoisoquinoline scaffold was synthesized. The molecular structure of **WN198** was confirmed by single-crystal X-ray diffraction analysis. **WN198** displays a strong DNA interaction and kept a topoisomerase I inhibitory activity as detected by in vitro tests.

The compound exerts excellent cytotoxic activities against five adenocarcinoma cancer cell lines at a lower concentration compared to other classical topoisomerase drugs used in chemotherapies. It is particularly efficient against MDA-MB-231 (triple-negative breast cancer) cell line proliferation with an IC_50_ of 0.37 μM. The IC_50_ on non-cancerous cell line MCF-10A is significantly high compared to other copper complexes as topoisomerase inhibitors and no toxicity was detected below 5 µM for *Xenopus* oocyte maturation and embryo development.

**WN198** appears to be a new efficient drug to counteract cancer cells at low doses. **WN198** could benefit patients overexpressing topoisomerases, sensitize cancer cells to DNA-damaging chemotherapies [63], bypass unwanted side effects, or be part of synthetic lethality or synergic strategies.

## Figures and Tables

**Table 1:** Cell viability IC_50_ (in μM).

	MCF-7	MDA-MB-231	HeLa	HT-29	DU-145
**WN191**	0.58 ± 0.02	1.12 ± 0.01	0.80 ± 0.09	0.53 ± 0.01	1.09 ± 0.05
**WN198**	0.89 ± 0.22	0.37 ± 0.04	0.72 ± 0.06	1.06 ± 0.02	1.04 ± 0.34
**WN170**	0.46 ± 0.17	0.875 ± 0.01	0.630 ± 0.09	0.479 ± 0.07	0.305 ± 0.04
Cisplatin	40.396 ± 11.9	33.802 ± 1.27	19.287 ± 5.323	21.313 ± 7.475	2.308 ± 0.04
*Statistical difference* (**WN191**/**WN198**)	****	****	***	****	*

Data are expressed as the mean ± SD of three independent experiments. Statistics were based on the Student’s *t*-test of the difference between **WN191** and **WN198**. * *p* < 0.05, *** *p* < 0.005, and **** *p* < 0.001. Cisplatin and **WN170** were used as positive controls.

**Table 2:** Melting curves and fluorescence measurements for **WN191** and **WN198**.

Compound	Kapp (10^7^ M^−1^)	EtBr Displacement
**WN191**	8.964 ± 0.964	91%
**WN198**	10.791 ± 1.638	89%

Apparent binding constants were measured by fluorescence using [EtBr]/[DNA] = 1.26. Data are the mean of at least three independent experiments.

**Table 3:** Crystal data and structure refinement for Cu complex **WN198**.

Empirical Formula	C_25_H_34_Cl_2_CuN_4_O_12_
Formula weight	714.98
Temperature/K	296.15
Crystal system	Triclinic
Space group	P-1
a/Å	9.0523(12)
b/Å	11.6041(14)
c/Å	14.3430(19)
α/°	89.459(8)
β/°	87.775(8)
γ/°	87.345(9)
Volume/Å^3^	1503.8(3)
Z	2
ρ_calc_g/cm^3^	1.579
μ/mm^−1^	0.972
F(000)	738.0
Crystal size/mm^3^	0.213 × 0.177 × 0.034
Radiation	MoKα (λ = 0.71073)
2Θ range for data collection/°	2.842 to 51.992
Index ranges	−11 ≤ h ≤ 12, −15 ≤ k ≤ 15, −19 ≤ l ≤ 19
Reflections collected	24167
Independent reflections	5754 [R_int_ = 0.0575, R_sigma_ = 0.0819]
Data/restraints/parameters	5754/3/350
Goodness-of-fit on F^2^	1.028
Final R indexes [I ≥ 2σ (I)]	R_1_ = 0.1042, wR_2_ = 0.2914
Final R indexes [all data]	R_1_ = 0.1547, wR_2_ = 0.3380
Largest diff. peak/hole/e Å^−3^	1.49/−1.39

## Data Availability

Data are contained within the article.
